# Cannabinoid and nicotine exposure during adolescence induces sex-specific effects on anxiety- and reward-related behaviors during adulthood

**DOI:** 10.1371/journal.pone.0211346

**Published:** 2019-01-31

**Authors:** Anna N. Pushkin, Angeline J. Eugene, Valeria Lallai, Alan Torres-Mendoza, J. P. Fowler, Edison Chen, Christie D. Fowler

**Affiliations:** Department of Neurobiology and Behavior, University of California, Irvine, Irvine, CA, United States of America; Radboud University Medical Centre, NETHERLANDS

## Abstract

Nicotine and cannabis use during adolescence has the potential to induce long lasting changes on affective and cognitive function. Here, we examined whether adolescent exposure to nicotine, the cannabinoid agonist WIN55-212,2 (WIN), or co-exposure to both would alter operant learning, locomotion, and anxiety- and reward-related behaviors in male and female mice during adulthood. Males exposed to a moderate dose of WIN (2 mg/kg) or co-exposed to nicotine and the moderate dose of WIN exhibited decreased anxiety-associated behaviors and increased cognitive flexibility, but did not differ in operant learning or generalized locomotion. In contrast, differences were not found among the females in these measures at the moderate WIN dose or in both sexes with exposure to a low WIN dose (0.2 mg/kg). Furthermore, a sex-dependent dissociative effect was found in natural reward consumption. Males exposed to the moderate dose of WIN or co-exposed to nicotine and the moderate dose of WIN demonstrated increased sucrose consumption. In contrast, females exposed to the moderate dose of WIN exhibited a decrease in sucrose consumption, which was ameliorated with co-administration of nicotine. Together, these novel findings demonstrate that adolescent exposure to cannabinoids in the presence or absence of nicotine results in altered affective and reward-related behaviors during adulthood.

## Introduction

Tobacco smoking results in millions of preventable deaths each year worldwide. Nicotine, the main psychoactive component in tobacco, is considered to be responsible for the development and maintenance of dependence in humans. Nicotine’s effects on adolescent development have become of increasing concern given the emergence of e-cigarettes, which deliver vaporized nicotine [1]. According to a nationwide CDC survey, ~30–45% of high school students self-reported prior use of cigarettes, vaporized nicotine products, and/or cannabis [2]. Given that legalization of recreational cannabis across states since the time of this survey, the number of adolescents exposed to this drug will likely continue to increase through both primary and second-hand exposure. Importantly, studies in humans examining co-use of these drugs have found that individuals who reported smoking both cannabis and tobacco cigarettes consumed more cigarettes than those using tobacco alone [3]. Furthermore, the practice of mulling (combining tobacco with cannabis to smoke as a joint) has been reported as frequently occurring in adolescent users, with high incidence (up to 90%) among daily cigarette smokers in some populations [4, 5]. Interestingly, chronic male cannabis users show decreased activation of the caudate nucleus in relation to reward anticipation as compared to nicotine users and non-smokers [6], suggesting altered function of reward-related circuitries dependent on prior drug exposure. Chronic use of cannabis during adolescence has also been linked to an elevated risk of psychosis, anxiety disorders, and depression [7]. For instance, Crane and colleagues found that symptoms of depression were positively correlated with both cannabis use and tobacco smoking frequency in male, but not female, subjects [8–10]. In contrast, Wright and colleagues report that cannabis use predicted increased depressive symptoms in both males and females, but increased anxiety symptoms and behavioral disinhibition were only found in females [9]. Adolescent substance users have also been found to exhibit abnormalities in brain function, structure, and volume [10]. However, given the nature of human studies, it is difficult to establish a causal link between early life exposure and the development of these conditions, especially as drug co-use is not often considered and may partially explain inconsistent findings noted in prior studies.

Nicotine acts in the brain via the neuronal nicotinic acetylcholine receptors, which are ligand-gated ion channels expressed on both presynaptic and postsynaptic membranes [11, 12]. Rodent models have shown that adolescent nicotine exposure alone may lead to behavioral alterations during adulthood. For instance, in male and female rats, adolescent nicotine enhances nicotine reward and intake during adulthood [13, 14]. Nicotine during adolescence has also been shown to increase depression-associated behaviors, decrease exploratory activity, and induce deficits in context conditioning to shock-associated cues in adult rats [15–17]. However, in these studies, differences were not found with anxiety-associated behaviors, extinction of contextual conditioning, or cued fear responses [15–17]. In mice, sex dependent effects have been noted, with adolescent nicotine consumption leading to decreased anxiety-associated behaviors in adult females, but not males [18]. With regard to cannabinoids, Δ^9^-tetrahydrocannabinol (THC) has been classified as the main psychoactive component in cannabis and exerts its actions on cannabinoid 1 (CB1) and cannabinoid 2 (CB2) receptors in the brain and periphery. Differential patterns of expression for these receptors are found across adolescent development and between males and females, and notably CB1 receptors exhibit the highest level of expression during the developmental period of mid-adolescence [19, 20]. Following THC administration in adolescence, adult female, but not male, rats exhibit depression-associated behaviors, but no changes in anxiety-associated or general locomotor behaviors were observed [21]. Interestingly, the depression-associated behavioral effects found in females were paralleled by significantly reduced CB1 receptor expression and activity in the amygdala, ventral tegmental area and nucleus accumbens, whereas similar changes were not found in the ventral tegmental area and nucleus accumbens of males [21]. Further, administration of WIN 55,212–2, a CB1 and CB2 specific agonist, during adolescence has similarly been shown to increase depressive-like behaviors, as well as palatable food intake, during adulthood in male rats [22, 23]. Together, these prior findings demonstrate that early life exposure to either nicotine or cannabinoid agonists alone can alter later affective and cognitive function, which introduces the possibility of potential synergistic or opposing effects under co-use conditions.

In the current studies, we sought to examine whether nicotine and cannabinoid co-exposure during mid-adolescence would result in altered affective and reward-seeking behavior during adulthood. While prior studies have examined each drug and/or behavioral measure independently, the current investigations represent the first study of a co-exposure condition, which is commonly found in human subjects, and the resulting effects on multiple cognitive and affective measures. To this end, adolescent mice were exposed to the cannabinoid receptor agonist, WIN55,212–2, and/or nicotine and then assessed for cognitive, anxiety-related and depression-related behaviors during adulthood. Drug exposure occurred during postnatal day 38–49, which corresponds to mid-adolescence in rodents or ~13–17 years of age in humans [19, 24]. Based on prior evidence of differential responses for males and females with drug-related effects and baseline receptor expression [7, 19, 25], male and female mice were examined in a within-sex manner. Further, given that significant differences were found in behavioral measures at the moderate dose of the cannabinoid agonist, a second study was then conducted to examine whether these effects would be maintained with a lower dose of the cannabinoid agonist. Together, these studies provide evidence that adolescent drug exposure alters affective and reward-related behaviors during adulthood in a sex- and drug-dependent manner.

## Materials and methods

### Animals

Male and female wildtype C57BL/6J mice were derived from breeders in our laboratory animal facilities. Mice were maintained in an environmentally controlled vivarium on a 12 h reversed light/dark cycle. Food and water were provided *ad libitum* until behavioral training commenced. During food training, subjects were mildly food restricted to 85–90% of their free-feeding bodyweight, and water was provided *ad libitum*. Following food training and the lever reversal task, food and water were again provided *ad libitum* for at least 5 days prior to subsequent behavioral assessments. All experiments were conducted in strict accordance with the NIH Guide for the Care and Use of Laboratory Animals and were approved by the Institutional Animal Care and Use Committee at the University of California, Irvine.

### Drugs

The cannabinoid receptor agonist WIN55,212–2 mesylate (Tocris/Bio-Techne Corp, Minneapolis, ME, USA) was dissolved in vehicle containing 1% DMSO, 1% Tween-80, and 98% saline (sterile 0.9% NaCl). The doses of WIN55,212–2 administered were 2 or 0.2 mg/kg intraperitoneally (i.p.). The moderate dose of WIN (2 mg/kg) was selected based on prior studies demonstrating altered neural function with adolescent exposure in mice and rats [26, 27], and the low dose of WIN (0.2 mg/kg) was selected since this amount of drug has been shown to sustain daily reinforcing self-administration behavior in adolescent rats (~16 infusions/day at 0.0125 mg/kg/infusion = ~0.2 mg/kg per day) [28]. (-)-Nicotine hydrogen tartrate salt (MP Biomedicals, Santa Ana, CA, USA; 0215355491) was dissolved in 0.9% sterile saline and adjusted to pH 7.4. Nicotine was administered at a dose of 0.36 mg/kg, subcutaneous (s.c.) (free-base form); this dose is considered to be within the rewarding range of the dose response function that also elicits a behavioral response in adolescent C57BL/6J mice [29, 30]. Peripheral injections were administered at a volume of 10 mL/kg.

### Adolescent injection schedule

Beginning on postnatal day (PND) 38, the first groups of male and female mice were randomly subdivided into four experimental groups: (1) Control (saline s.c., vehicle i.p.), (2) NIC (0.36 mg/kg nicotine s.c., vehicle i.p.), (3) WIN (saline s.c., 2 mg/kg WIN i.p.), and (4) NIC/WIN (0.36 mg/kg nicotine s.c., 2 mg/kg WIN i.p.). Saline and vehicle were the solutions used to dissolve nicotine and WIN, respectively. Mice received once daily injections for 12 consecutive days from PND 38 to PND 49. The daily injection schedule was selected to model an experimental pattern of adolescent exposure. Body weight was recorded prior to each injection. The second study included mice treated as above, but they were subdivided into the following experimental groups: 1) Control (saline s.c., vehicle i.p.), (2) LdWIN (saline s.c., low dose (0.2 mg/kg) WIN i.p.), and (3) NIC/LdWIN (0.36 mg/kg nicotine s.c., 0.2 mg/kg WIN i.p.). For both studies, subjects were tested in multiple smaller cohorts to enhance rigor and reproducibility of the findings.

### Operant food training

On PND 70, subjects were mildly food restricted and trained to press a lever in an operant chamber (Med Associates, Fairfax, VT, USA) for food pellets (20 mg; TestDiet) under a fixed-ratio 5, time out 20 s (FR5TO20s) schedule of reinforcement. Each session was performed using 2 retractable levers (1 active, 1 inactive). Completion of the response criteria on the active lever resulted in the delivery of a food pellet. Responses on the inactive lever were recorded but had no scheduled consequences. Once stable responding was achieved (criteria >30 pellets per session across consecutive 3 sessions), the lever assignment was switched to examine cognitive flexibility. In the reversal task, the previous inactive lever became active, in that food pellets were earned in accordance with the established FR5TO20s schedule. In contrast, the previously active lever became inactive, in which responses were recorded but without scheduled consequence. All behavioral responses were automatically recorded by MedAssociates software.

### Open field locomotor test

The open-field chamber was composed of Plexiglas (35 cm L × 35 cm W × 31 cm H). After a 5-minute habituation period, subjects were scored in the open-field apparatus for a 15-minute test to assess locomotor activity. Activity was recorded with a video camera and scored by two experimenters blinded to the group condition with ANY-Maze Software (Stoelting Co., Wood Dale, IL, USA).

### Elevated plus maze

The elevated plus maze (EPM) was composed of 4 opaque runways 5 cm wide and 35 cm in length, which were elevated 40 cm from the floor. Two opposing closed runways had opaque walls 15 cm in height, whereas the other two opposing sides did not contain walls (open arms). Subjects were placed in the center portion of the elevated plus maze and behavior was recorded for 5 min with a video camera. Behavior was scored by two blinded experimenters with ANY-maze software.

### Sucrose consumption

Subjects were habituated to sucrose pellet consumption for 2 days prior to sucrose testing, during which time 60 mg of sucrose pellets (raspberry flavored; TestDiet, St. Louis, MO, USA) was provided for each subject in the home cage. On the third day, subjects were individually examined in home cage conditions, but were single housed and provided 200 mg of total sucrose pellets in a dish. All subjects were maintained under *ad libitum* full food conditions, and thus were not food restricted during testing. Sucrose eaten was recorded at specified intervals (5, 10, 15, 20, 30, 40, 50, 60 min) by experimenters blinded to the group condition. At the end of each session, experimenters examined the cage for breakage or disintegration of sucrose pellets; this occurred on only a few occasions and in these instances, the remnant amount was calculated and included in the final mg amount of sucrose remaining. Mice were required to consume at least one 20mg sucrose pellet within the first 30-min time period for inclusion in the study.

### Chow food consumption

Subjects were examined for their daily intake of mouse chow. Mice were restricted to daily feeding sessions of 6 hr periods. During these sessions, subjects were individually housed and provided full access to consume 6–8 grams of standard chow (LabDiet 5P76, TestDiet), and water was provided in the feed cages *ad libitum*. Food was weighed prior to and after each session. After 3 days of habituation to the feeding protocol, data were collected on the fourth day and analyzed across groups.

### Forced swim test

A cylindrical tank (22.5 cm diameter x 26 cm height) was filled with room temperature (23–25°C) water at a level of 15cm from the bottom of the tank. For testing, each subject was held by the tail, and slowly placed in the water. Mice were videotaped for the 5 min swim test duration. Data were quantified by experimenters blinded to the group assignment. Analysis of distance traveled was assessed with AnyMaze software, and the number and quantity of immobility bouts was hand scored by two separate experimenters to ensure accurate assessments.

### Statistical analyses

Given that these studies sought to investigate the effects of drug exposure relative to the control condition within each sex, statistical comparisons were performed separately for males and females based on this *a priori* hypothesis. Data were analyzed by a t-test, one-way or two-way ANOVA with Prism 7 software (GraphPad, La Jolla, CA, USA), as appropriate. Data obtain across sessions was analyzed with a repeated measures two-way ANOVA. Significant main or interaction effects were followed by Bonferroni post-hoc comparison with correction for multiple comparisons. The criterion for significance was set at α = 0.05.

## Results

### Experiment 1: Nicotine and Moderate Dose of WIN

#### Body weight during adolescent injections

**Fig 1:**
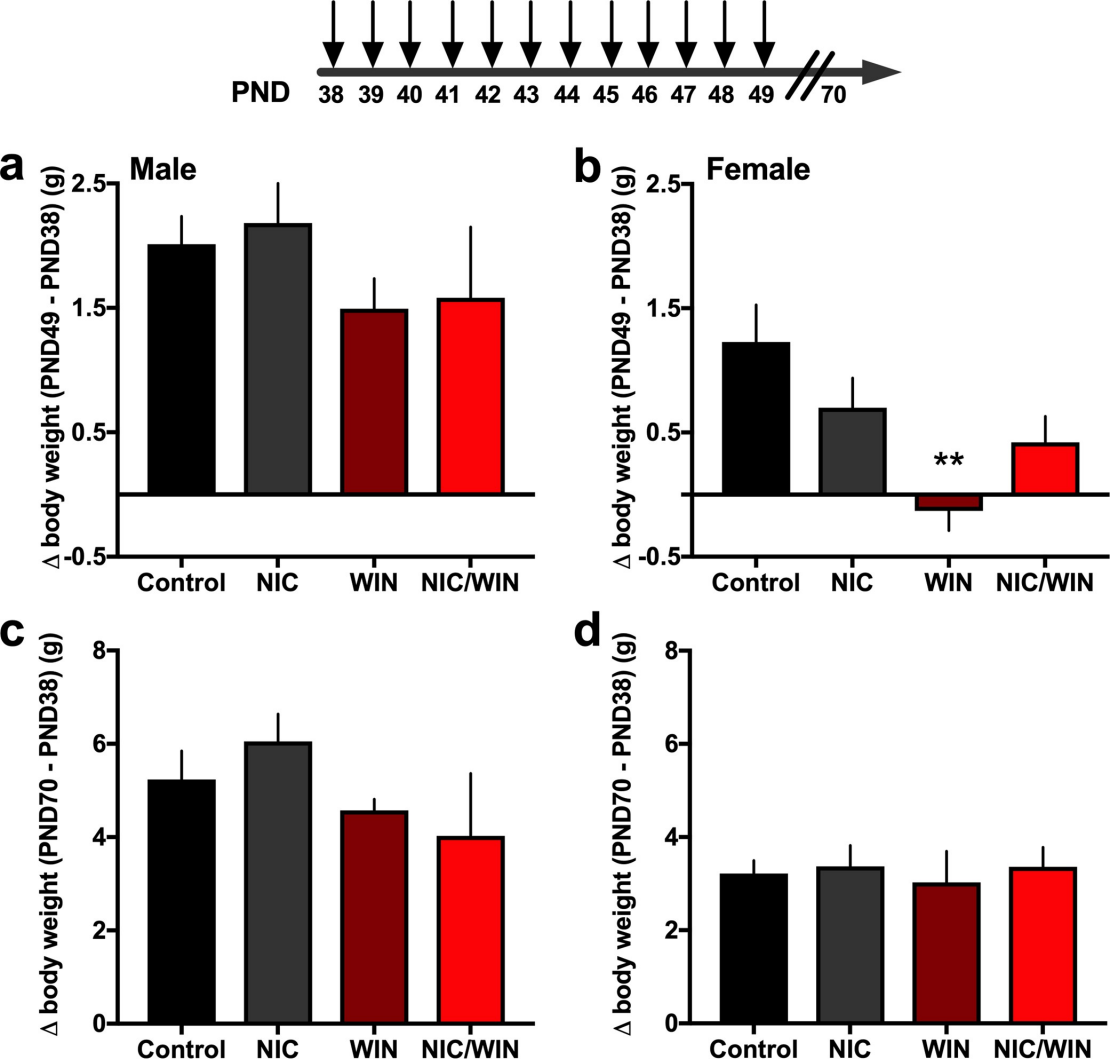
Adolescent drug exposure paradigm and change in body weight with nicotine and/or a moderate dose of the cannabinoid agonist, WIN. **(a)** Male mice (n = 5-8/group) were examined for their change in body weight from the first injection on PND 38 to the final day of the injection series on PND 49. Statistically significant group differences were not found. **(b)** Female mice (n = 6-12/group) were examined for their change in body weight from PND38 to PND 49, and a significant difference was found with the female WIN-treated group exhibiting a decrease as compared to the control, and this effect was reversed under the co-exposure condition. **p<0.001 **(c)** During adulthood at PND 70, body weight differences were again not found based on adolescent drug exposure in males. **(d)** Females from all groups exhibited a similar increase in body weight when assessed on PND 70. Control: saline and vehicle injection group; NIC: nicotine and vehicle injection group; WIN: saline and 2 mg/kg WIN-55,212–2 injection group; NIC/WIN: nicotine and 2 mg/kg WIN-55,212,2 injection group. Data represent mean values ± SEM.

In an initial cohort, we assessed whether drug condition would affect change in body weight during the duration of the drug injections from postnatal day (PND) 38 (day 1 injection) to PND 49 (day 12 injection) (Fig 1). Change in body weight was also compared to adulthood at PND70, prior to the commencement of behavioral assessments. Groups did not differ in body weight at PND 38 following random group assignment. For males, group differences were not found when comparing the change in body weight from PND 38 to PND 49 (Fig 1A) (*One-way ANOVA*, F_(3,20)_ = 0.91, p = 0.455) or to PND 70 (Fig 1C) (*One-way ANOVA*, F_(3,20)_ = 1.536, p = 0.236). In contrast, female subjects exhibited a statistically significant difference in body weight change at PND 49 (Fig 1B) (*One-way ANOVA*, F_(3,29)_ = 4.27, p = 0.013), with post-hoc tests revealing a decrease in body weight for the WIN group compared to the control (p<0.001). However, these effects were ameliorated during the post-injection time period, as no significant differences among the groups were found at PND 70 (Fig 1D) (*One-way ANOVA*, F_(3,29)_ = 0.101, p = 0.959).

#### Operant learning

**Fig 2:**
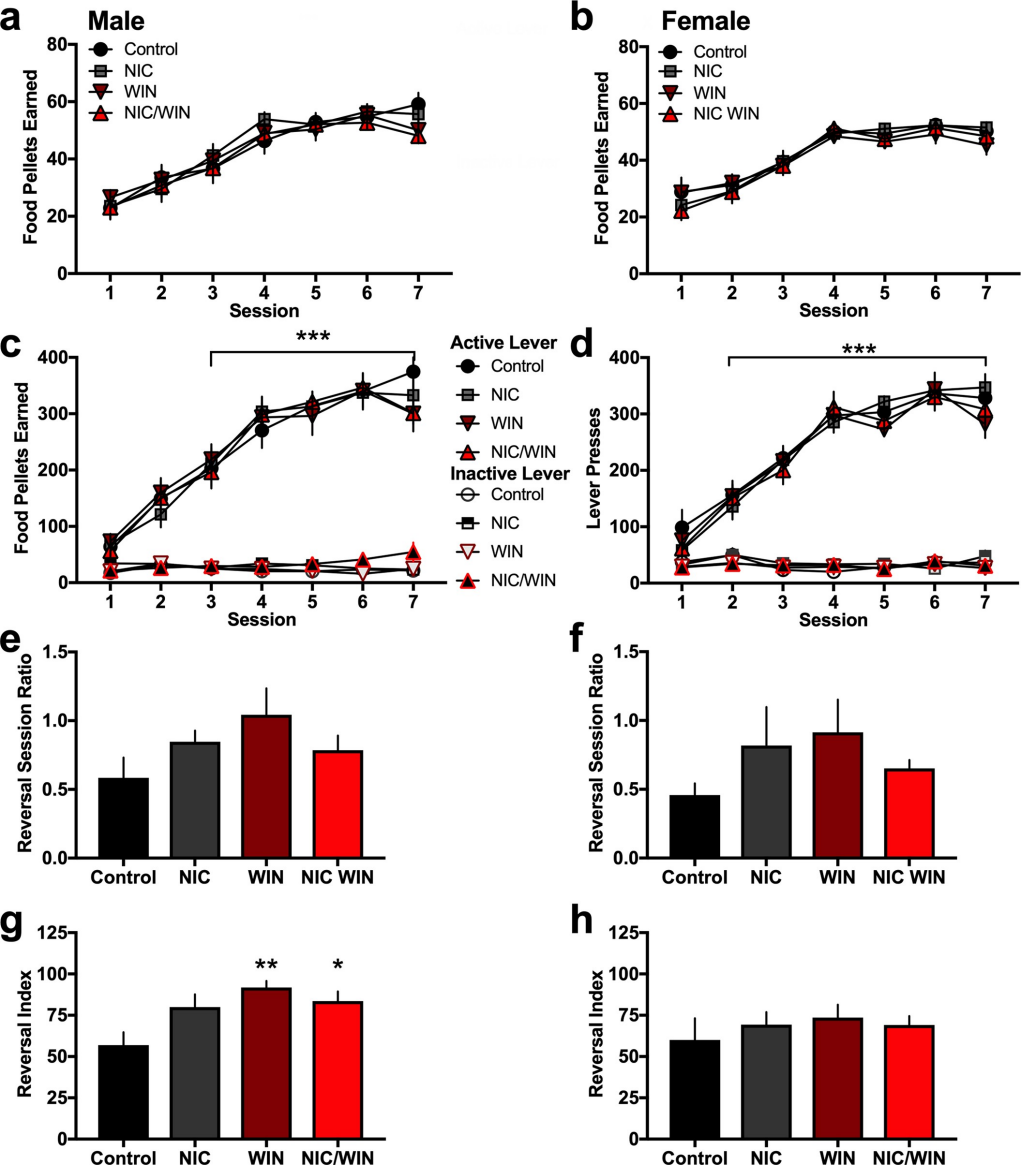
Operant learning and cognitive flexibility following adolescent exposure to nicotine and/or a moderate dose of the cannabinoid agonist in adult mice. **(a)** Male mice (n = 9-10/group) were examined for their ability to learn an operant task to obtain food reward. Groups did not differ in their number of food pellets earned across sessions. **(b)** Female mice (n = 7-9/group) were examined with operant food training, and differences were not found among groups in the number of food pellets earned across sessions. **(c)** The number of active and inactive lever presses was examined across sessions for all groups. Significant main and interaction effects were found with all groups exhibiting statistically significant preference for the active lever versus the inactive lever for sessions 3–7. ***p<0.0001 **(d)** Female mice also exhibited significant main and interaction effects for all groups when comparing number of active to number of inactive lever presses for sessions 2–7. ***p<0.0001 **(e-h)** In the cognitive flexibility assessment, mice were required to reverse their lever pressing behavior for the active and inactive lever. During the reversal session, the ratio of the number of active to inactive lever presses was derived (number active/number inactive). The reversal index was also calculated as a comparison to the baseline day of responding prior to the lever switch ((number active reversal session/number active baseline session)*100). **(e)** Male mice did not exhibit any group differences in the active:inactive ratio. **(f)** Female mice also did not exhibit any group differences in the active:inactive ratio. **(g)** For the reversal index, the male WIN and NIC/WIN groups exhibited increased lever pressing behavior on the reversal session, as evidenced by the higher reversal index for these groups compared to the control. **p<0.01 **(h)** Female mice did not exhibit any group differences in the reversal index. Data represent mean values ± SEM.

Groups were examined for their ability to learn an operant task to respond for food reward. All exposure groups exhibited similar learning curves in earning food pellets for both males (Fig 2A) (*Repeated measures two-way ANOVA*, Group: F_(3,33)_ = 0.26, p = 0.853; Session: F_(6,198)_ = 68.02, p<0.0001; Interaction: F_(18,198)_ = 0.78, p = 0.721) and females (Fig 2B) (*Repeated measures two-way ANOVA*, Group: F_(3,30)_ = 0.29, p = 0.835; Session: F_(6,180)_ = 79.7, p<0.0001; Interaction: F_(18,180)_ = 0.73, p = 0.783). When comparing active and inactive lever pressing, all groups exhibited a clear dissociation between the active and inactive lever consistent with learned behavior in the operant task. For males, significant main and interaction effects were found (Fig 2C) (*Repeated measures two-way ANOVA*, Group: F_(7,66)_ = 71.86, p<0.0001; Session: F_(6,396)_ = 93.39, p<0.0001; Interaction: F_(42,396)_ = 13.62, p<0.0001). For females, similar differences were also found (Fig 2D) (*Repeated measures two-way ANOVA*, Group: F_(7,60)_ = 105.2, p<0.0001; Session: F_(6,360)_ = 128.1, p<0.0001; Interaction: F_(42,360)_ = 20.51, p<0.0001). For both males and females, post-hoc tests revealed significant differences between the number of active and inactive lever presses for all groups from sessions 3–7, but the groups did not differ from one another when comparing responding among drug conditions on each lever.

After establishing consistent responding on the active lever, cognitive flexibility was examined in the reversal task. Subjects were required to switch their lever pressing behavior, as the active and inactive lever assignments were reversed. Interestingly, the groups did not differ during the reversal session for their total number of rewards earned (males, one-*way ANOVA*, F_(3,33)_ = 1.86, p = 0.156; females, one-*way ANOVA*, F_(3,30)_ = 0.32, p = 0.814) or for the within-session active to inactive lever pressing ratio for both males (Fig 2E) (*One-way ANOVA*, F_(3,33)_ = 1.88, p = 0.153) and females (Fig 2F) (*One-way ANOVA*, F_(3,30)_ = 0.92, p = 0.443). Groups also did not differ in the latency to respond on the active lever for males (*One-way ANOVA*, F_(3,32)_ = 0.35, p = 0.787) and females (*One-way ANOVA*, F_(3,30)_ = 0.25, p = 0.861). Next, we obtained a reversal index, which was derived by the equation: ((number of active lever presses during the reversal session)/(number of active lever presses during the baseline session prior to reversal))*100. Surprisingly, the male WIN and NIC/WIN groups exhibited a higher reversal index, indicating greater food reward seeking behavior under conditions of higher cognitive demand (Fig 2G) (*One-way ANOVA*, F_(3,33)_ = 5.19, p = 0.004). In contrast, differences in the reversal index were not found among the female groups (Fig 2H) (*One-way ANOVA*, F_(3,30)_ = 0.41, p = 0.748).

#### Locomotion

**Fig 3:**
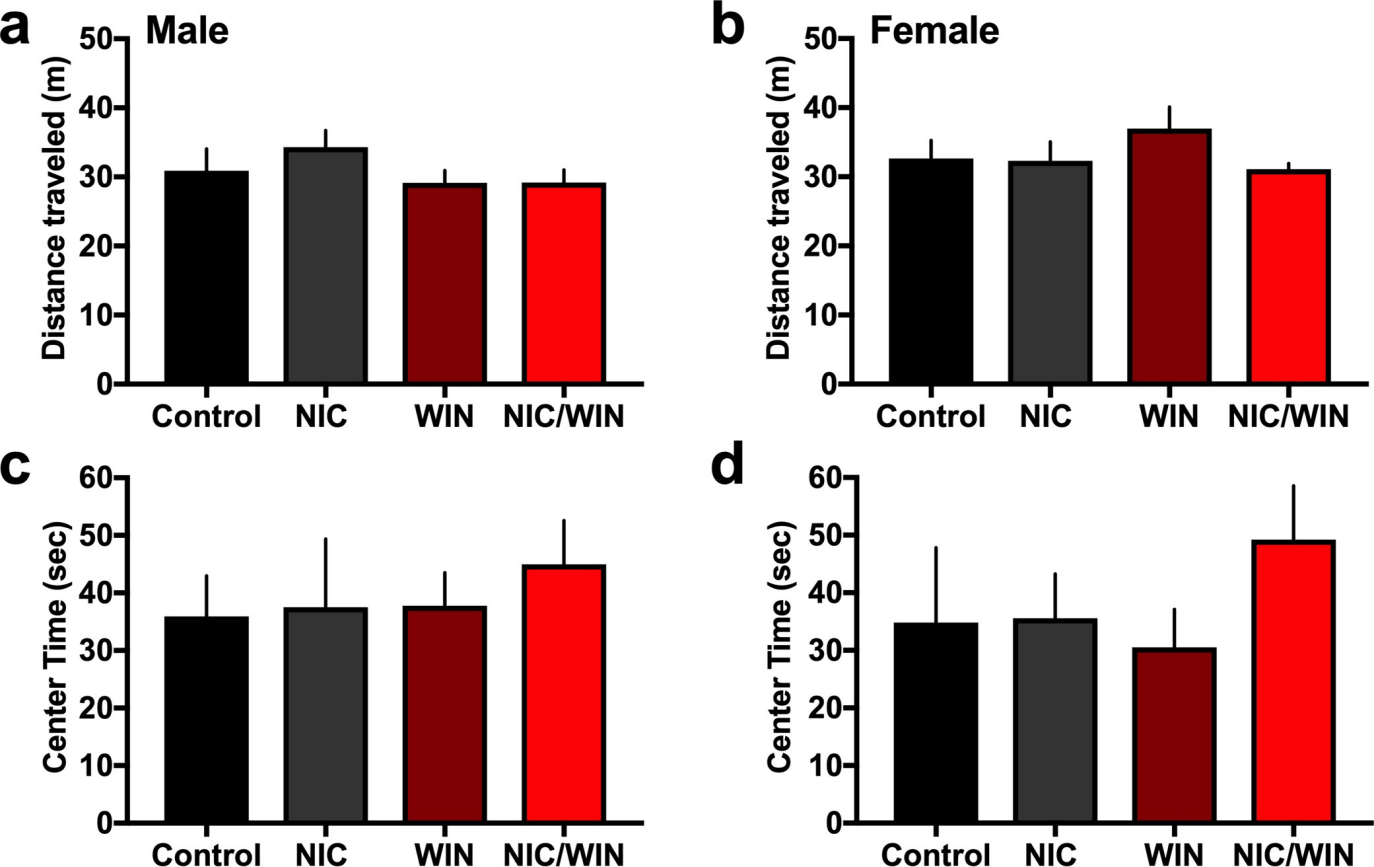
Adolescent nicotine and/or cannabinoid agonist exposure does not alter locomotor behavior during adulthood. **(a)** Male mice (n = 8-12/group) did not differ in the distance travelled in the open field. **(b)** Female mice (n = 6-10/group) did not differ in the distance travelled in the open field. **(c)** Analysis of the time spent in the center of the field did not reveal any differences in male subjects. **(d)** Females also did not differ in the time spent in the center of the field. Data represent mean values ± SEM.

The open field test was utilized to assess generalized locomotion and exploratory behavior. No statistically significant differences were observed among drug conditions in distance travelled for males (Fig 3A) (*One-way ANOVA*, F_(3,35)_ = 1.13, p = 0.351) and females (Fig 3B) (*One-way ANOVA*, F_(3,27)_ = 0.90, p = 0.456). Further, for the duration of time spent in the center of the open field, no differences were found among groups for males (Fig 3C) (*One-way ANOVA*, F_(3,35)_ = 0.17, p = 0.918) and females (Fig 3D) (*One-way ANOVA*, F_(3,27)_ = 0.71, p = 0.553).

#### Anxiety-related assessment

**Fig 4:**
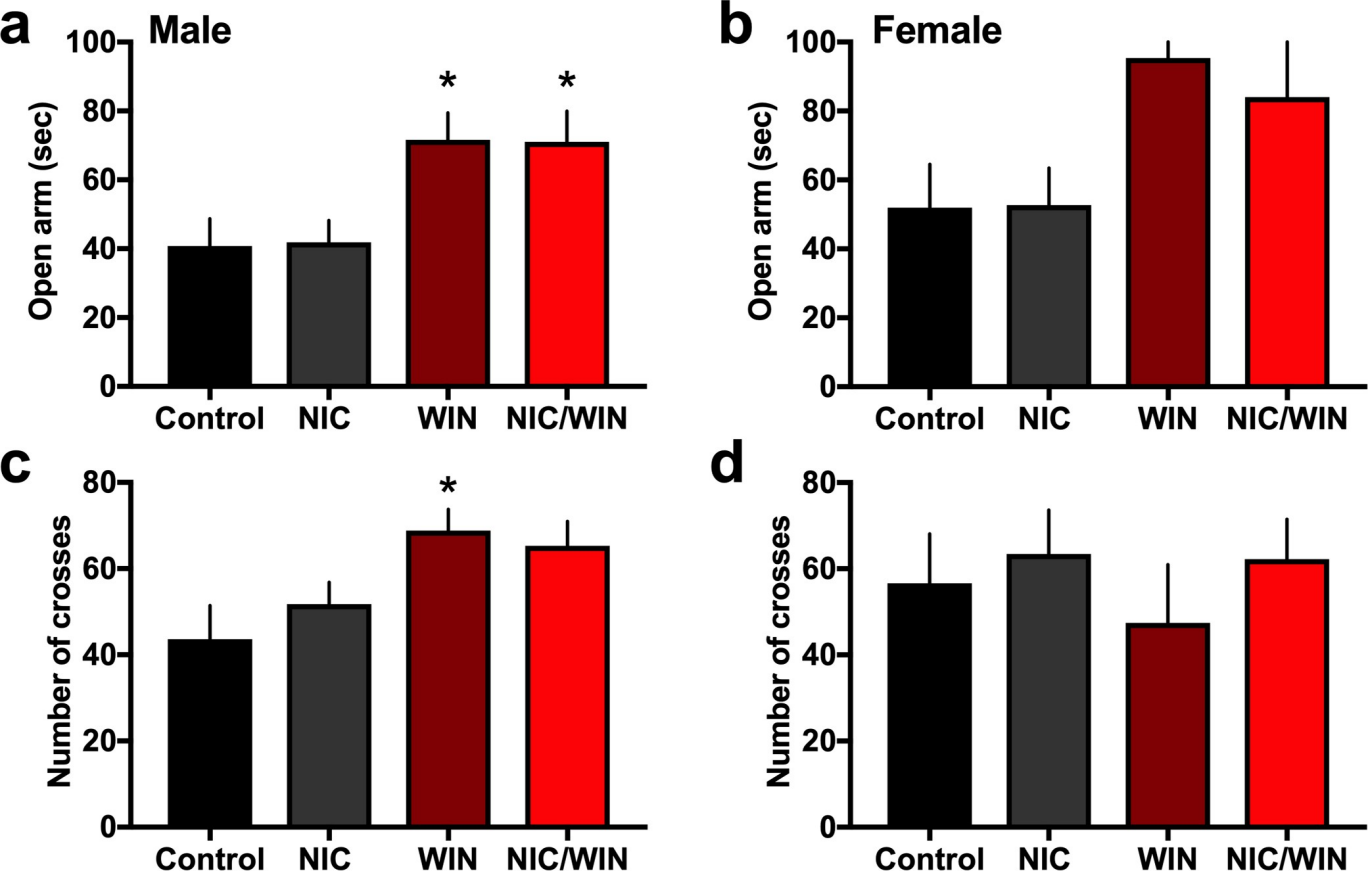
Altered anxiety-related behavior in male, but not female, adult mice following adolescent cannabinoid agonist exposure at a moderate dose. **(a)** Male mice (n = 7-8/group) exhibited differential responding in the elevated plus maze dependent on adolescent drug exposure. Specifically, mice treated with the cannabinoid agonist WIN or co-treated with WIN and nicotine exhibited increased time on the open arm, indicating a decrease in anxiety-related behavior. *p<0.05 **(b)** Female mice (n = 5-7/group) did not exhibit any statistically significant differences in the elevated plus maze open arm time. **(c)** For the male mice, the WIN group also displayed a significant increase in the number of crosses between arms compared to the control group, potentially indicative of increased exploratory behavior, an effect which was decreased with NIC/WIN co-exposure. *p<0.05 **(d)** The number of arm crosses did not differ significantly between the groups for the female mice. Data represent mean values ± SEM.

As a measure for anxiety-related behavior, subjects were assessed in the elevated plus maze, in which increased time in the open arms is thought to represent an anxiolytic effect. In the males, we found a significant increase in the time spent in the open arm of the elevated plus maze for both the WIN and NIC/WIN groups as compared to the control condition (Fig 4A) (*One-way ANOVA*, F_(3,26)_ = 5.00, p = 0.007). Interestingly, the WIN only group also exhibited an increase in the number of crosses between the arms (Fig 4C) (*One-way ANOVA*, F_(3,26)_ = 3.72, p = 0.024), and this was likely indicative of decreased anxiety related effects and/or increased exploratory behavior, rather than an overall increase in general locomotion given the absence of effects in the above noted open field test. Moreover, the presence of nicotine with WIN resulted in no significant difference from the control condition, and thus, the co-exposure condition counteracted the WIN-induced increase in exploratory behavior. In contrast, differences among groups were not found for females in the open arm time (Fig 4B) (*One-way ANOVA*, F_(3,18)_ = 0.70, p = 0.565) or number of arm crosses (Fig 4D) (*One-way ANOVA*, F_(3,18)_ = 0.43, p = 0.737).

#### Sucrose and food consumption

**Fig 5:**
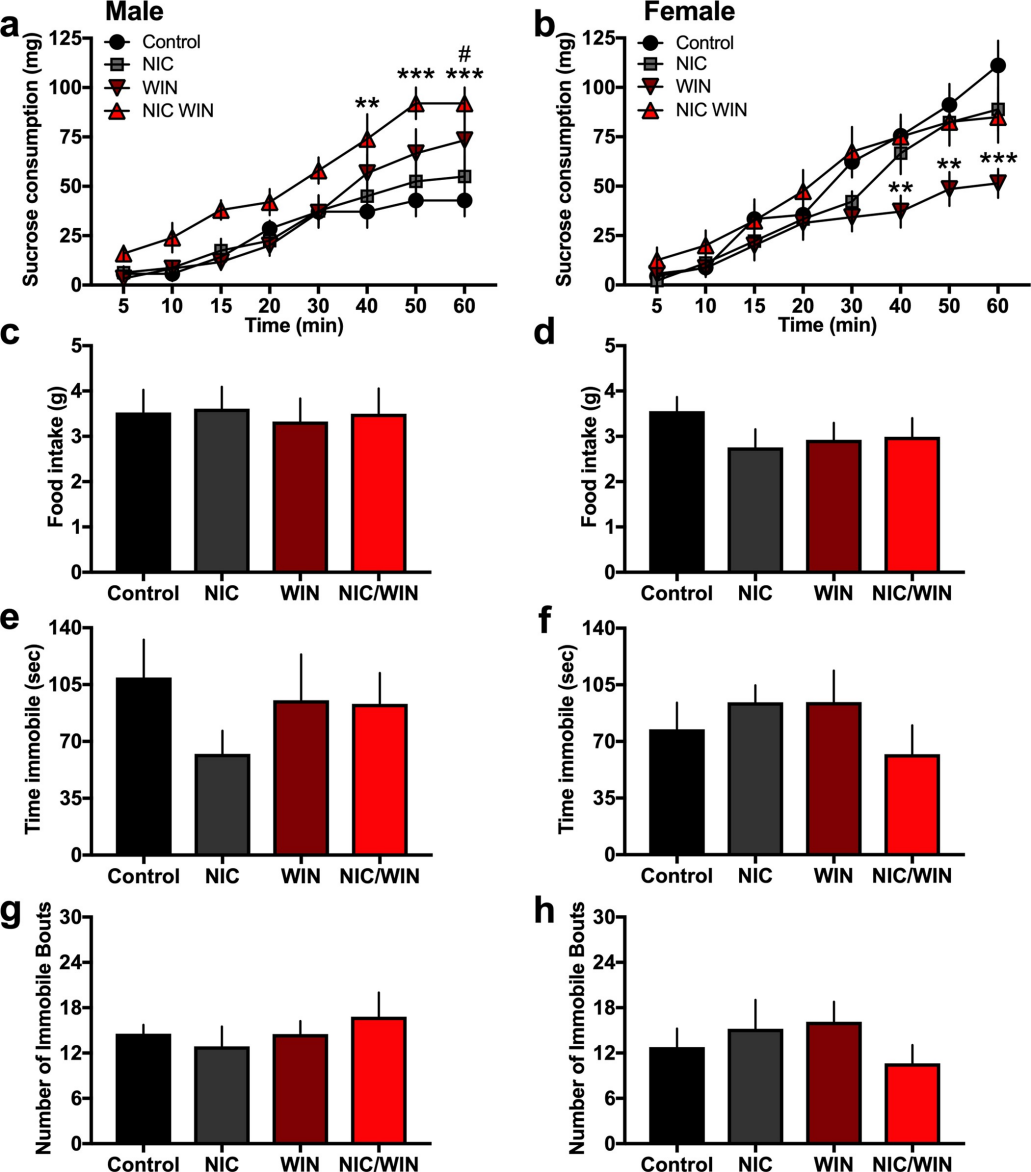
Within sex-specific effects in natural reward consumption, but not other depression-associated behaviors, in adult mice following adolescent exposure to nicotine and/or a moderate dose of WIN. **(a)** Male subjects (n = 5-8/group) were examined for cumulative sucrose consumption during a 1 hr test. The NIC/WIN mice exhibited a significant increase in sucrose consumption at the 40, 50 and 60 min time points, as compared to the control group. **p<0.01, ***p<0.001 Further, the WIN group consumed greater sucrose than the control group at the 60 min time point. #p<0.05 **(b)** In contrast, female mice (n = 7-9/group) exhibited a differential effect, with the WIN treated group consuming less sucrose pellets than the control group at the 40, 50 and 60 min time points. **p<0.01, ***p<0.001 **(c-d)** Since sucrose consumption could be secondary to generalized food intake among groups, subjects were examined for standard chow intake during a restricted 6hr daily feeding period. Male groups did not differ in chow food intake **(c)**, nor did the female groups **(d)**. **(e-h)** To examine whether the sucrose consumption findings were consistent with other measures of depression-associated behaviors, the forced swim test was employed. Groups did not differ in the time immobile for both males **(e)** and females **(f)**. Similarly, groups did not differ in the number of immobile bouts for both males **(g)** and females **(h)**. Data represent mean values ± SEM.

Mice were examined for their consummatory behavior of natural reward with 1hr access to sucrose pellets. Statistically significant main and interaction effects were found for the amount of sucrose consumed across groups in males (Fig 5A) (*Two-way ANOVA*, Group: F_(3,22)_ = 3.71, p = 0.027; Time: F_(7,154)_ = 67.54, p<0.0001; Interaction: F_(21,154)_ = 1.85, p = 0.018). Post-hoc analysis revealed a significant increase in the NIC/WIN group compared to the control group at the 40, 50 and 60 min time points (p<0.01, p<0.001, p<0.001, respectively), and a significant increase for the WIN group compared to the control group at the 60 min time point (p<0.05). Female subjects also exhibited significant group differences (Fig 5B) (*Two-way ANOVA*, Group: F_(3,29)_ = 2.16, p = 0.115; Time: F_(7,203)_ = 111.5, p<0.0001; Interaction: F_(21,203)_ = 3.35, p<0.001), with the post-hoc analysis revealing a decrease in consumption for the WIN group relative to the control group at time points 40, 50 and 60 min (p<0.01, p<0.01, p<0.0001, respectively). Differences were not found among groups for the initial latency to consume a sucrose pellet (males, one-*way ANOVA*, F_(3,23)_ = 0.11, p = 0.99; females, one-*way ANOVA*, F_(3,29)_ = 0.43, p = 0.733). To ensure that the sucrose consumption was not secondary to general food intake among the groups, subjects were also assessed for their daily consumption of mouse chow. Male groups did not differ in the amount of food consumed (one-*way ANOVA*, F_(3,30)_ = 0.31, p = 0.82) (Fig 5C), nor did the females groups (one-*way ANOVA*, F_(3,28)_ = 2.41, p = 0.09) (Fig 5D).

#### Depression-associated behavior

To further determine whether the differences in sucrose consumption were due to reward related effects, as predicted, or secondary to an anhedonia/depression-associated state, we next examined swim behavior in the forced swim test. In this assessment, we found no significant differences among groups in the time immobile or number of immobile bouts for both males (Fig 5E and 5G, respectively) (Time immobile: *One-way ANOVA*, F_(3,22)_ = 1.01, p = 0.409; Immobile bouts: *One-way ANOVA*, F_(3,22)_ = 0.472, p = 0.705) and females (Fig 5F and 5H, respectively) (Time immobile: *One-way ANOVA*, F_(3,29)_ = 0.91, p = 0.450; Immobile bouts: *One-way ANOVA*, F_(3,29)_ = 0.66, p = 0.57).

### Experiment 2: Nicotine and Low Dose of WIN

#### Body weight during adolescent injections

**Fig 6:**
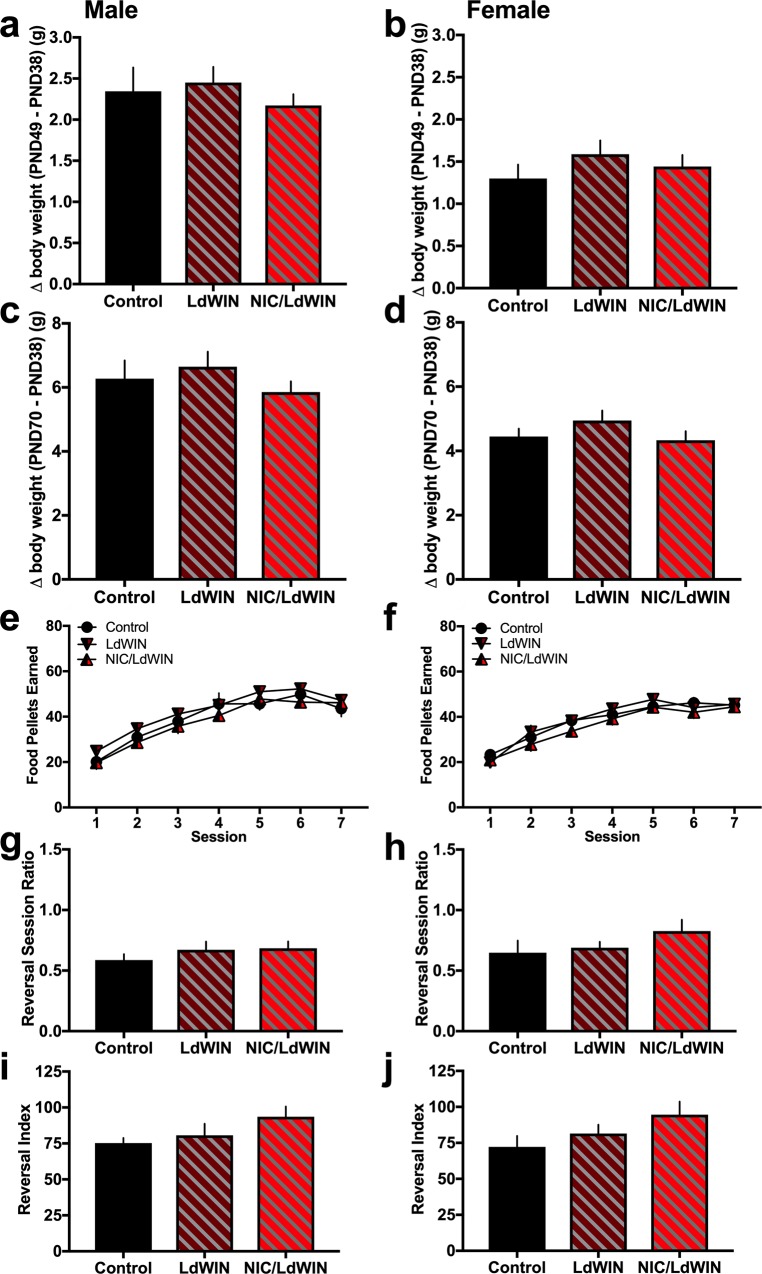
Body weight change, operant learning and cognitive flexibility following low dose exposure to the cannabinoid agonist, with or without nicotine. **(a)** Male mice (n = 9-16/group) were examined for their change in body weight from the first injection on PND 38 to the final day of the injection series on PND 49. Statistically significant group differences were not found. **(b)** Female mice (n = 13-17/group) were examined for their change in body weight from PND38 to PND 49, and no significant differences were found. **(c-d)** During adulthood at PND 70, differences in body weight were again not found across adolescent drug exposure conditions for either males **(c)** or females **(d)**. **(e)** Male mice (n = 14-16/group) across groups did not differ in their ability to learn an operant task to obtain food reward. **(f)** Female mice (n = 14-16/group) also did not differ in their operant responding for food reward. **(g-j)** In the cognitive flexibility assessment, mice were next required to reverse their lever pressing behavior for the active and inactive lever. During the reversal session, the ratio of the number of active to inactive lever presses was derived; differences among groups were not found for both males **(g)** and females **(h)**. The reversal index was also calculated as a comparison to the baseline day of responding prior to the lever switch. **(i)** Male mice did not exhibit any group differences and equally switched their lever pressing behavior to the active lever. **(j)** Female mice also exhibited similar indices of cognitive flexibility in switching their behavior to respond on the reassigned active lever. Control: saline and vehicle injection group; LdWIN: saline and 0.2 mg/kg WIN-55,212–2 injection group; NIC/LdWIN: nicotine and 0.2 mg/kg WIN-55,212,2 injection group. Data represent mean values ± SEM.

Similar to above, we first assessed whether drug administration would alter body weight during the duration of the drug injections from postnatal day (PND) 38 (day 1 injection) to PND 49 (day 12 injection) or during adulthood at PND70 (Fig 6). For males, group differences were not found when comparing the change in body weight from PND 38 to PND 49 (Fig 6A) (*One-way ANOVA*, F_(2,37)_ = 0.60, p = 0.555) or to PND 70 (Fig 6C) (*One-way ANOVA*, F_(2, 37)_ = 0.89, p = 0.419). Female subjects also did not exhibit differences in body weight change at PND 49 (Fig 6B) (*One-way ANOVA*, F_(2,42)_ = 0.83, p = 0.444) or at PND 70 (Fig 6D) (*One-way ANOVA*, F_(2,42)_ = 1.37, p = 0.265).

#### Operant learning

Groups were next examined for their ability to learn an operant task to respond for food reward. All exposure groups exhibited similar learning curves in earning food pellets for both males (Fig 6E) (*Repeated measures two-way ANOVA*, Group: F_(2,41)_ = 1.27, p = 0.30; Session: F_(6,246)_ = 84.69, p<0.0001; Interaction: F_(12,246)_ = 0.68, p = 0.77) and females (Fig 6F) (*Repeated measures two-way ANOVA*, Group: F_(2,43)_ = 0.79, p = 0.460; Session: F_(6,258)_ = 86.37, p<0.0001; Interaction: F_(12,258)_ = 0.84, p = 0.607). After establishing consistent responding on the active lever, cognitive flexibility was examined in the reversal task. Subjects were required to switch their lever pressing behavior, as the active and inactive lever assignments were reversed. Interestingly, the groups did not differ during the reversal session for the within-session active to inactive lever pressing ratio for both males (Fig 6G) (*One-way ANOVA*, F_(2,33)_ = 0.56, p = 0.576) and females (Fig 6H) (*One-way ANOVA*, F_(2,38)_ = 1.29, p = 0.288). Next, we obtained the reversal index as described above, and no significant differences were found for males (Fig 6I) (*One-way ANOVA*, F_(2,33)_ = 1.56, p = 0.225) and females (Fig 6J) (*One-way ANOVA*, F_(2,38)_ = 1.97, p = 0.154).

#### Locomotion and anxiety-related behaviors

**Fig 7:**
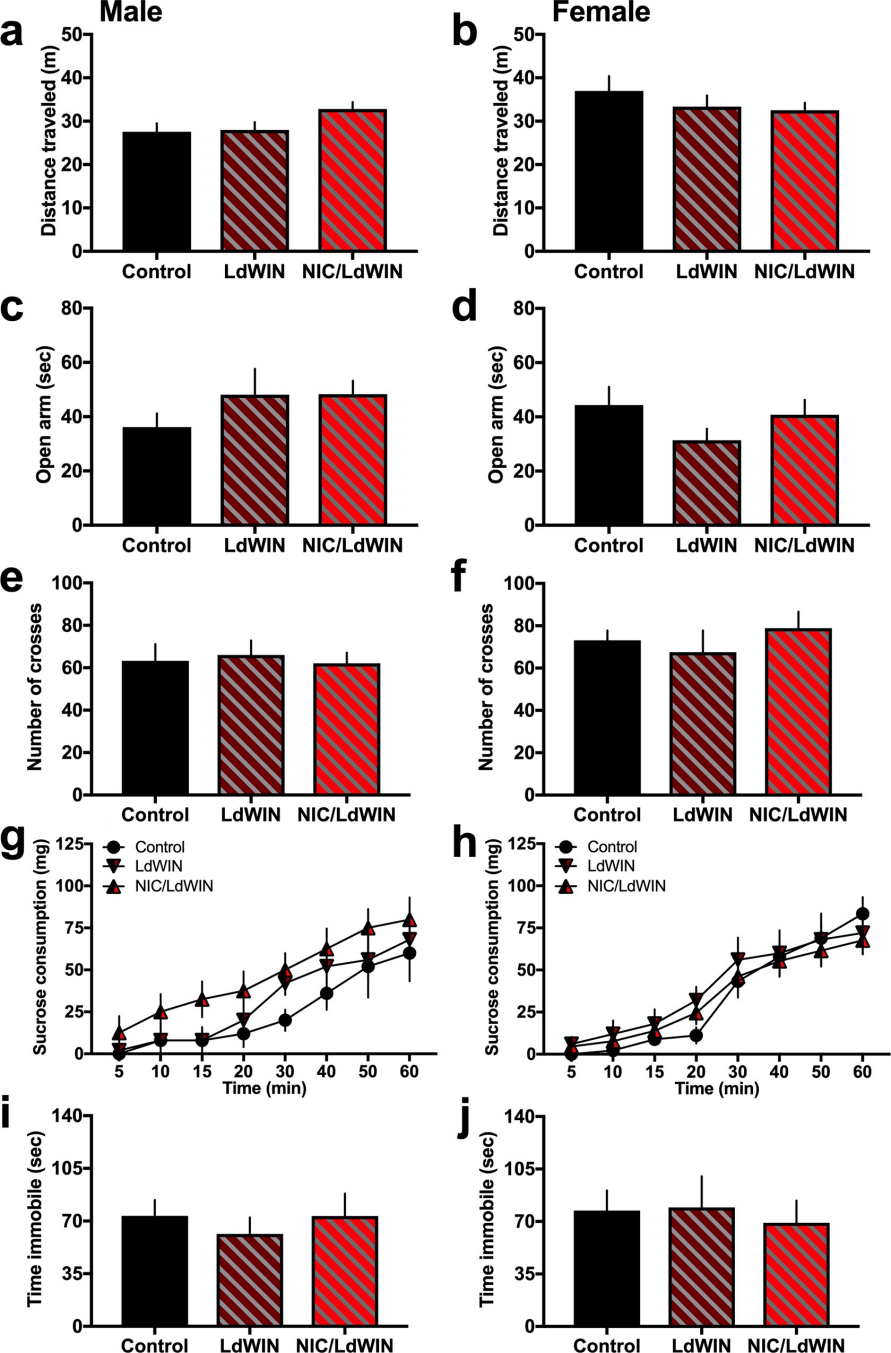
Adolescent low dose cannabinoid agonist exposure with or without nicotine does not alter locomotor or affective-associated behaviors during adulthood. **(a)** Male mice (n = 10-12/group) did not differ in the distance travelled in the open field. **(b)** Female mice (n = 11-13/group) did not differ in the distance travelled in the open field. **(c-f)** To assess anxiety-associated behaviors, mice were then tested in the elevated plus maze. Statistically significant differences were not found among the groups in the time spent on the open arms for males **(c)** and females **(d)**. Differences were also not found in the number of arm crosses for males **(e)** and females **(f)**. **(g-j)** Reward related and depression-associated behaviors were then assessed across groups; differences were not found for sucrose consumption in males **(g)** and females **(h)**, nor were differences found in the forced swim test for males **(i)** and females **(j)**. Data represent mean values ± SEM.

The open field test was utilized to assess generalized locomotion and exploratory behavior in the low dose WIN groups. No statistically significant differences were observed among drug conditions for distance travelled in males (Fig 7A) (*One-way ANOVA*, F_(2,29)_ = 2.50, p = 0.099) and females (Fig 7B) (*One-way ANOVA*, F_(2,33)_ = 0.82, p = 0.451). Further, for the duration of time spent in the center of the open field, no differences were found among groups in males (*One-way ANOVA*, F_(2,29)_ = 0.05, p = 0.946) and females (*One-way ANOVA*, F_(2,33)_ = 0.24, p = 0.789). Subjects were then assessed in the elevated plus maze to examine anxiety-related and exploratory behaviors. In the males, significant differences among the groups were not found in the time spent in the open arm of the elevated plus maze (Fig 7C) (*One-way ANOVA*, F_(2,28)_ = 0.96, p = 0.395). Similarly, differences among groups were not found for females in the open arm time (Fig 7D) (*One-way ANOVA*, F_(2, 32)_ = 1.36, p = 0.271). With regard to the number of crosses in the elevated plus maze, differences were not present among the groups for males (Fig 7E) (*One-way ANOVA*, F_(2,28)_ = 0.09, p = 0.914) and females (Fig 7F) (*One-way ANOVA*, F_(2, 32)_ = 0.52, p = 0.598).

#### Reward and depression-associated behaviors

Groups exposed to the low dose of WIN were examined for their consummatory behavior of natural reward with 1hr access to sucrose pellets. Treatment groups did not differ for sucrose consumption in both males (Fig 7G) (*Two-way ANOVA*, Group: F_(2,20)_ = 1.89, p = 0.182; Time: F_(7,140)_ = 39.67, p<0.0001; Interaction: F_(14,140)_ = 0.49, p = 0.933) and females (Fig 7H) (*Two-way ANOVA*, Group: F_(2,29)_ = 0.24, p = 0.79; Time: F_(7,203)_ = 67.32, p<0.0001; Interaction: F_(14,203)_ = 0.79, p = 0.677). Finally, we examined for depression-associated behavior in the forced swim test, but no statistically significant differences were found in the time immobile for both males (Fig 7I) (*One-way ANOVA*, F_(2,25)_ = 0.33, p = 0.721) and females (Fig 7J) (*One-way ANOVA*, F_(2,30)_ = 0.11, p = 0.893).

## Discussion

Given the growing incidence of nicotine and cannabis experimentation during adolescence, we sought to examine whether such exposure would lead to altered behavioral effects during adulthood. In these studies, we found that male adolescent exposure to a moderate dose of the cannabinoid receptor agonist, WIN55,212–2 (WIN), led to increased cognitive flexibility in a learning reversal task, decreased anxiety-associated behaviors, and increased natural reward consumption, but no differences in general locomotor or depression-related behavior. Interestingly, the co-exposure condition of both nicotine and the moderate dose of WIN led to similar behavioral profiles as WIN alone in these measures, suggesting that a potentiative or additive effect was not present for these behaviors. However, with regard to the number of lane crosses in the elevated plus maze, the nicotine and WIN co-exposure condition appeared to exert a counteractive effect on the WIN-induced increase in exploratory behavior at the moderate dose, suggesting an opposing effect with adolescent exposure to both drugs. With regard to females, the moderate dose of WIN induced a lower body weight during the adolescent period, but co-exposure with nicotine appeared to exert an opposing effect that resulted in no difference from the control condition. However, these effects of WIN on body weight were transitory, as the difference in females did not persist into adulthood. For the behavioral assessments, female subjects were overall more resistant to the long-term effects of adolescent drug exposure. Group differences were only found in the sucrose consumption test, in which the moderate dose WIN females exhibited decreased natural reward consumption compared to the control females. However, differences from the control were not found with the female nicotine and WIN co-exposure condition for sucrose consumption, suggesting that the presence of nicotine ameliorated the actions of WIN on reward circuitry during the adolescent period. In contrast, adolescent exposure to a low dose of WIN had no effect on physiological or behavioral measures, either alone or in the presence of nicotine, for both males and females. Taken together, these findings demonstrate that while adolescent cannabinoid agonist exposure at a moderate dose exerts variable effects on both physiological and behavioral measures in males and females, co-administration of nicotine surprisingly counteracted some of these effects by normalizing to control levels.

While prior studies have examined the effects of adolescent exposure of either nicotine or WIN alone on later behaviors, the current findings represent the first examination of the effects of co-exposure during mid-adolescence and subsequent long-term effects on adult behavior. This age range was selected based on the correlation to human adolescence with higher levels of experimentation and more recurrent patterns of drug consumption than that found in younger individuals. With regard to nicotine alone, opposing effects have been found in male Sprague-Dawley rats with increased depression-associated behaviors, but no difference in anxiety-associated behaviors, during adulthood [15]. However, these behavioral differences were only found at higher nicotine doses approximately twice that administered in the current study. Chronic exposure approaches with a minipump or nicotine patch at higher doses (≥5 mg/kg/day) have also demonstrated decreased exploratory activity, decreased food consumption under anxiety-related conditions, and deficits in contextual condition to shock-associated cues in Sprague-Dawley rats [16, 17]. In mice, adolescent exposure to high dose minipump (12 mg/kg/day) has also been shown to disrupt contextual fear condition, but not cued fear conditioning [31]. However, since studies have shown that of those adolescents age 12–17 who smoke, the majority smoke one or less than one cigarette per day (50.1%)[32], the current studies focused on a rewarding dose with once daily exposure as an investigative goal. Thus, the lack of difference in the behavioral measures with nicotine exposure in the current studies may be attributed to this relatively lower dose administered. Along these lines, it should be noted that this dose was selected based on the rewarding effects of doses in this range, as assessed with the brain reward threshold measure [29], and behavioral effects elicited in adolescent mice [30], and thus, the current results have particular relevance to experimental patterns of drug consumption found in youth.

With adolescent cannabinoid agonist exposure, findings derived from prior rat studies have been somewhat variable. In one study, adolescent male and female rats treated with the cannabinoid agonist, CP 55,940, exhibited overall increased time on the open-arm of the elevated plus maze, but these effects were not maintained when examining males and females independently [33], suggesting these differences may have been confounded by baseline differences between the sexes. Since CP 55,940 has high affinity for both the CB1 and CB2 receptors, as well as GPR55, the lack of differences within each sex for drug condition may also have been due to actions on alternate signaling pathways or differences in agonist actions. Interestingly, male Sprague-Dawley rats treated with WIN, the CB1 and CB2 specific agonist, during adolescence exhibited increased depressive-like behaviors in the forced swim and sucrose consumption tests [22, 23]. In our mouse studies, we did not find any differences in these measures with the low dose of WIN and opposing effects at the moderate dose of WIN, indicating that species differences in metabolism and/or genetic heritability factors likely mediate the effects of cannabinoids on adolescent neurodevelopment. Finally, adolescent WIN exposure has also been found to increase palatable food intake and alter attribution of incentive salience for food reward in adult male Long Evans rats [23]. The increase in natural reward-related effects with adolescent exposure is consistent with our findings at the moderate WIN dose in mice, suggesting cannabinoid exposure during adolescence similarly alters brain reward pathways to enhance subsequent responsiveness to natural reward. Interestingly, Schoch and colleagues also demonstrated increased expression of the endocannabinoids anandamide and oleoylethanolamine in the nucleus accumbens only during a food restricted state with adolescent WIN exposure in rats [23]. Thus, dependent on the availability of food and level of satiety, changes in neural systems regulating reward-related behaviors may be differentially affected in the presence of cannabinoids. Along these lines, it is interesting to note that in the current study, mice were at a satiated level (not food restricted) during sucrose consumption, during which time the opposing differences were found in males and females exposed to adolescent WIN. However, during conditions of food restriction, such as during operant food training in the current study, group differences only emerged for males in the reversal task. Thus, altered endocannabinoid signaling may account for this effect during the food restricted state, whereas other mechanisms likely underlie the behavioral differences observed in the anxiety and natural reward-related measures.

Cannabinoid and nicotinic acetylcholine receptors exhibit overlapping expression within brain regions implicated in reward-related and affective behaviors, including the prefrontal cortex, ventral tegmental area, nucleus accumbens, medial habenula, interpeduncular nucleus and hippocampus [7, 34]. On the cellular level, both receptors types are expressed on presynaptic terminals and function to modulate release of various neurotransmitters. For instance, with acute administration, both drugs increase extracellular dopamine in the nucleus accumbens and prefrontal cortex [35, 36], and adolescent cannabinoid or nicotine exposure have also been shown to affect cholinergic, serotonergic and noradrenergic signaling mechanisms [22, 31, 37]. Thus, in consideration of the effects of nicotine and cannabinoids on several neurotransmitter systems and the behavioral findings from the current studies, future studies will need to dissect the differential impact of single or co-drug exposure during adolescence on neural signaling mechanisms.

In conclusion, activation of cannabinoid receptors with or without nicotine led to differential sex-specific effects on anxiety- and reward-related behaviors during adulthood. Together, these studies provide evidence that adolescent exposure to drugs of abuse may lead to alterations in affective and cognitive behaviors during adulthood. These data support the conclusion that consumption of cannabis by youth may alter later cognitive function, and thus, policy approaches should be considered to discourage and/or restrict substance use by this vulnerable population.
